# Seasonal Variation in Raw Milk VOC Profile within Intensive Feeding Systems

**DOI:** 10.3390/foods12091871

**Published:** 2023-04-30

**Authors:** Carmela Zacometti, Alessandra Tata, Andrea Massaro, Giorgia Riuzzi, Marco Bragolusi, Giulio Cozzi, Roberto Piro, Sara Khazzar, Gabriele Gerardi, Flaviana Gottardo, Severino Segato

**Affiliations:** 1 Experimental Chemistry Laboratory, Istituto Zooprofilattico Sperimentale delle Venezie, 36100 Vicenza, Italy; czacometti@izsvenezie.it (C.Z.); atata@izsvenezie.it (A.T.); amassaro@izsvenezie.it (A.M.); mbragolusi@izsvenezie.it (M.B.); rpiro@izsvenezie.it (R.P.); 2 Department of Animal Medicine, Production and Health, University of Padova, 35020 Legnaro, Italy; giorgia.riuzzi@studenti.unipd.it (G.R.); giulio.cozzi@unipd.it (G.C.); sara.khazzar@studenti.unipd.it (S.K.); gabriele.gerardi@unipd.it (G.G.); flaviana.gottardo@unipd.it (F.G.)

**Keywords:** volatile organic compounds, raw milk, milking season, interactive principal component analysis

## Abstract

The study aimed to assess the seasonal variation in raw milk volatile organic compounds (VOCs) from three indoor feeding systems based on maize silage (*n* = 31), silages/hay (*n* = 19) or hay (*n* = 16). After headspace solid-phase microextraction (HS-SPME), VOC profiles were determined by gas chromatography (GC). Chemical and VOC (log_10_ transformations of the peak areas) data were submitted to a two-way ANOVA to assess the feeding system (FS) and season (S) effects; an interactive principal component analysis (iPCA) was also performed. The interaction FS × S was never significant. The FS showed the highest (*p* < 0.05) protein and casein content for hay-milk samples, while it did not affect any VOCs. Winter milk had higher (*p* < 0.05) proportions of protein, casein, fat and some carboxylic acids, while summer milk was higher (*p* < 0.05) in urea and 2-pentanol and methyl aldehydes. The iPCA confirmed a seasonal spatial separation. Carboxylic acids might generate from incomplete esterification in the mammary gland and/or milk lipolytic activity, while aldehydes seemed to be correlated with endogenous lipid or amino acid oxidation and/or feed transfer. The outcomes suggested that VOCs could be an operative support to trace raw milk for further mild processing.

## 1. Introduction

Milk is a multiphase and polydisperse fluid with a wide combination of chemical constituents that mainly reflects the metabolic footprint of cows’ feeding systems, and especially of type and storage method of the forage portion of the diet [1]. Different analytical methods, based on metabolomic fingerprinting and, in some cases, quantification of low molecular weight compounds, can be applied in raw milk traceability [2], including nuclear magnetic resonance (NMR) spectroscopy, liquid chromatography/mass spectrometry (LC/MS) and isotope ratio mass spectrometry (IRMS) [3]. These methods present different sensitivities and a varying metabolite coverage. Even though NMR remains the most commonly used analytical platform in milk and cheese traceability [4,5], IRMS is an advanced technique used to authenticate raw milk and to identify altitude (δ^2^H and δ^18^O), type of grazing vegetation (δ^15^N) or animal feed (δ^13^C) [6]. While NMR has a low sensitivity and measures compounds in micromolar/millimolar concentrations, chromatographic based techniques can identify metabolites at nanomolar to picomolar concentrations, allowing a much higher number of small metabolites to be detected [3]. Among micro-compounds, the native volatile organic compounds (VOCs), analysed by gas chromatography (GC), contribute to the flavour and sensory profile of milk, defining the so-called “terroir” of the food, a key authenticity trait used to discriminate high quality-labelled dairy products, such as protected designation of origin (PDO) cheese [7,8]. Indeed, forage feeding and its closely related factors, such as the botanical composition, the phenological stage at harvest and the storage method (i.e., silage vs. dried forage), have been reported to influence both the VOC profile and sensory traits of dairy products [9]. So far, the effect of the feeding system on the volatile and sensory properties of cow milk has been mainly focused on the comparison between pasture grazing and total mixed ration (TMR), which proved significantly higher contents of acetic acid, pentanol and toluene in milk from the grass-based pasture [10], as well as changes in many milk metabolites [11]. The replacement of a maize silage-based TMR with grass or a mix of grass and clover pasture resulted in a wide change in the volatile profile of cow raw milk, with TMR-milk containing more ethyl and methyl esters and aldehydes and pasture-milk containing more hydrocarbons and sulphur compounds [12]. In another study, the transition of cows’ feeding from ensiled forage to pasture grazing led to a significant decrease of some methyl ketones (2-tridecanone and 2-pentadecanone) in milk, and a prolonged grazing time increased the presence of diterpenoids such as 1-phytene and 2-phytene [13]. These latter diterpenoids derived from the hydrogenation of neophytadiene that, in turn, generates from phytol, which is an intermediate of the ruminal degradation of chlorophyll [13], even if phytol can be converted also in phytanic acid [14]. 

To the best of our knowledge, a comparison between the effects of indoor TMR diets based on maize silage or permanent meadow hay on native VOCs in raw milk is still lacking [15]. Furthermore, there is a low amount of available literature data about the effects of the milking season on the VOC profile of milk from dairy cows kept indoors year-round and fed TMR based on dried or ensiled forage crops from arable land (e.g., maize, ryegrass, lucerne, clovers). This type of dairy farm is predominant in the Po Valley, the nest of Italian top-quality cheeses (e.g., Parmigiano Reggiano, Grana Padano, Asiago, Provolone, etc.) as well as in the main milk and dairy producing countries around Europe. Despite the fact that cow’s bulk milk from indoor dairy system is considered to keep almost the same characteristics all year around, the milk and cheese chemical composition, fatty acids (FA) and VOC profiles can change according to the main roughage components of the TMR (e.g., forage maturation stage, storage method) across the milking season [16,17]. Moreover, a recent study [18] revealed that small organic acids were the predominant milk VOCs in autumn, while spring milk had higher contents of alcohols, esters and ketones. As recent developments show a tendency toward the production of dairy products from raw milk, there is a need to decode the whole milk flavour offering the possibility for further quality optimisation by using mild technologies [19]. Therefore, the assessment of the impact of annual season on VOCs in milk from different indoor feeding systems (FS) could contribute to the better traceability of milk’s organoleptic-sensory footprint. In fact, studies under on-farm conditions are highly relevant for the selection of effective forage management practices to improve the quality of labelled raw milk-based dairy products. To this end, the main target of this study was to evaluate the effects of the production season on the volatile profile of raw milk in three indoor FS based on silage and/or dried forage. 

## 2. Materials and Methods

### 2.1. Experimental Design, Sample Collection and Chemical Analysis

The study protocol did not interfere with the routine farm activities or management decisions; only bulk milk samples were collected after the farm owners’ permission was received and in accordance with guidelines given by the veterinarians involved. Since there was no manipulation of the animals, the impact of the study on the health and welfare of the cows was negligible, making ethics committee approval unnecessary. The experimental design involved 17 dairy farms located in a lowland area of north-eastern Italy (45°42′ lat. N; 11°38′ long. E). All farms reared high-producing Holstein and Brown dairy cows in loose housed barns with cubicles. The dairy farms were sampled to be representative of comparable herd characteristics in terms of parity (2.7 lactations on average), days in milk (DIM, 183 d on average), milk and fat protein corrected milk (FPCM) yield (29.7 kg d^−1^ on average), thus the main distinctive difference among them was the forage component of the feeding systems.

The raw milk sampling collection was carried out in the winter of 2019–2020 and in the following summer. The three studied indoor FS differed in the forage base of the total mixed ration (TMR) used: (i) maize silage and grass silages (ensiled maize and grass, EMAG); (ii) a mix of maize and cereal winter silages and grass haylage (ensiled and haylage forage, EAHF); (iii) hay from permanent meadow and grassland (meadow and grass hay, MAGH). Average feed compositions of the rations for the three FS fed to the lactating cows during the cold (winter) and warm (summer) seasons and their proximate composition on a dry matter (DM) basis are reported in (Table 1). The TMR diets were formulated estimating the energy and nutrient requirements of the lactating cows and processed in the mixer wagon following the mixing time and order of adding ingredients suggested by the feeding machinery manufacturers. Cows were fed once a day and TMR diet was available for ad libutum intake on each participating farm. Samples of TMR were collected at each milk sampling and immediately analysed for DM and proximate composition according to the AOAC procedures as described in detail in our previous studies [20]. The (amylase) neutral detergent fiber (aNDF), acid detergent fiber (ADF) and acid detergent lignin (ADL) fractions were determined by an Ankom Fiber Analyzer (ANKOM Technology Corporation, Fairport, NY, USA). The aNDF was analysed with sodium sulphite, heat-stable alpha-amylase, F57 bags with 25 μm pore size (reagents and materials from ANKOM Technology Corporation, Fairport, NY, USA) and included residual ash [21], non-sequential ADF was evaluated according to Vogel et al. 1999 [22] and ADL (sulphuric acid lignin) using an Ankom-technology procedure [23]. 

Raw bulk milk samples were collected twice from each farm in both winter (December and February) and summer (June and August). According to the sampling protocol, a total of 66 raw milk samples were collected: 31 (16 winter and 15 summer), 19 (9 winter and 10 summer) and 16 (8 winter and 8 summer) for EMAG, EAHF and MAGH, respectively. On each investigated farm, the cows were milked twice a day in dedicated milking parlours approximately at 5 to 7 a.m. and 5 p.m.; the evening milk was stored at 4 (±0.5) °C overnight into refrigerated tanks (average volume of 5000 L), to which the morning milk was then added and agitated before sampling in Pyrex bottles with a Teflon cup and immediately delivered (at 4 °C) to the laboratories for chemical and VOC analysis. Collection, refrigeration, transport and storage of milk samples were standardised to minimise differences among farms and collection dates. Milk composition (protein, casein, fat, lactose) and chemical traits (pH, β-hydroxybutyrate, urea) were analysed by a Fourier-transform mid infrared (FT-MIR) spectroscopic technique by a MilkoScan FT6000 (Foss Electric A/S, Hillerød, Denmark). The somatic cell count (SCC, cells/mL of milk) was determined by a Fossomatic 5000 (Foss Electric A/S) and reported as [log_2_ (SCC/100,000) + 3].

### 2.2. HS-SPME and GC-FID Procedures 

Milk VOCs were extracted by headspace (HS) coupled to solid-phase microextraction (SPME). In details, 5 mL aliquot of the bulk milk samples were transferred into a headspace vial with aluminium cap and PTFE septum (Agilent Technologies, Santa Clara, CA, USA). An amount of 3.5 g of sodium chloride (NaCl, ≥99.0%, purchased from Sigma Aldrich, Steinheim, Germany) was added to each sample to enhance the volatilisation of the volatile molecules. The vial was placed on a magnetic stirrer for 45 min at 45 °C, with a sampling fiber coated with divinylbenzene/carboxen/polydimethylsiloxane (DVB/CAR/PDMS, stableflex, 50/30 μm, Merck KGaA, Darmstadt, Germany) in a holder suspended above. The DVB/CAR/PDMS fiber can adsorb both polar and non-polar compounds. Afterwards, the SPME fiber was withdrawn and inserted into the inlet of the gas chromatograph instrument equipped with a flame ionization detector (FID). The desorption of the VOCs from the SPME fiber was carried out for 90 min at 45 °C in the injection port of the gas chromatograph in split less mode. The pre-desorption and post-desorption conditioning were carried out for 10 min at 250 °C. The analyses of raw milk VOCs were carried out with a gas chromatograph 7890B (Agilent Technologies, Santa Clara, CA, USA) following the Agilent application note 5994-0546EN [24]. The application note describes in details the analytical method and the annotation of flavour and fragrance compounds based. Briefly, peak separation was carried out with a 30 m × 250 μm I × 0.25 μm HP-5ms column (Agilent Technologies, Santa Clara, CA, USA). The oven temperature, initially held at 60 °C for 3 min, was ramped to 240 °C at a heating rate of 3 °C min^−1^, and then was finally held at 240 °C for 3 min (60 min analysis time). Helium was used as carrier gas at a pressure of 13.4 psi with a constant flow of 1.46 mL min^−1^ to give a retention time of 32.000 min for n-hexadecane. The method was retention-time-locked using n-hexadecane as the locking standard. The FID signal was utilised for the semi-quantitative evaluation of the peak heights, as relative concentrations area units, of the chromatographically separated compounds. The MassHunter software (Agilent Technologies, Santa Clara, CA, USA) was used to calculate retention times, integrate the peaks and interrogate the compound library NIST 2017 (NIST, National Institute of Standards and Technology), and the Agilent flavour and fragrance retention time locked (RTL) library [25]. Each sample was analysed in duplicate and the area of each peak averaged.

### 2.3. Statistical Analysis 

Milk analytical data were statistically processed using RStudio software (v4.0.2; R Core Team 2022). MetaboAnalyst 5.0 web platform was used for comprehensive and integrative metabolomics of VOC data analysis [26]. A two-way ANOVA was performed to assess if the fixed effects of feeding system (FS) and season (S) and their interactions affected raw milk composition and the VOC profile. Since the majority of the VOC data (area under peak curves) were not normally distributed, they were log-transformed (log_10_ of the peak area) prior to ANOVA. A Bonferroni-adjusted significance test for pairwise comparisons among LS-means of FS was performed if FS was significant. 

An interactive principal component analysis (iPCA) was carried out to reveal the pattern of VOCs correlated to the three feeding systems (EAHF, EMAG, MAGH) and the two seasons (summer vs. winter). iPCA plots allowed visualization of the data using colours or shapes based on the different levels of interactions within groups. Only the significant (*p* < 0.05) variables were retrieved.

## 3. Results

### 3.1. Feed and Chemical Composition of Dietary Feeding Systems

Seasonal changes in the TMR ingredients were limited within the different FSs. However, in summer, there was a smaller replacement of maize silage with grass and cereal silages both in the EMAG and EAHF groups, but meadow hay was replaced by lucerne hay in MAGH (Table 1). The TMR had similar crude protein content among the FSs, whereas MAGH, based on hays as the main forage ingredients, was characterised by a slightly higher content of aNDF and a lower amount of starch, resulting in a higher aNDF:starch ratio, as compared to the two ensiled forage-based FSs (Table 1).

### 3.2. Milk Composition

There was no significant difference in raw milk constituents due to the FS × S interaction. Milk from the two ensiled forage-based feeding systems (maize silage vs. mix of silages) had significantly lower contents of protein and casein, whereas milk from cows fed hay-based TMR (MAGH) had a lower lactose content (Table 2). 

Milk pH, β-hydroxybutyrate and somatic cell count (as somatic cell score, SCS) were similar across feeding systems. The shift from winter to summer affected milk composition in all feeding systems, leading to a significantly lower protein, casein and fat contents in summer milk (Table 2). Moreover, significantly higher milk pH and urea values were measured in the summer than in the winter.

### 3.3. Milk Volatile Organic Compounds

The HS-SPME combined with GC-FID technique identified a total of 43 VOCs in the analysed milk samples (Supplementary Material, Table S1). These molecules included esters (12), carboxylic acids (8), alcohols (7), aldehydes (6), ketones (5), alkane (1), ether (1), and 3 compounds that could not be assigned. Consistent with the main milk chemical components, none of the VOCs was affected by a significant FS × S interaction. The comparison of FSs did not show any significant difference in the VOC profiles of raw milk. On the contrary, the season in which milk was produced had a significant effect on the abundance (expressed as log_10_ of the peak area) of many volatile compounds in the milk. Winter raw milk samples was characterised by a significantly higher content of four carboxylic acids (C3–C10), two aldehydes, 2-octanone as well as an unassigned compound detected at a retention time of 27.68 min (Table 3). 

**Figure 1:**
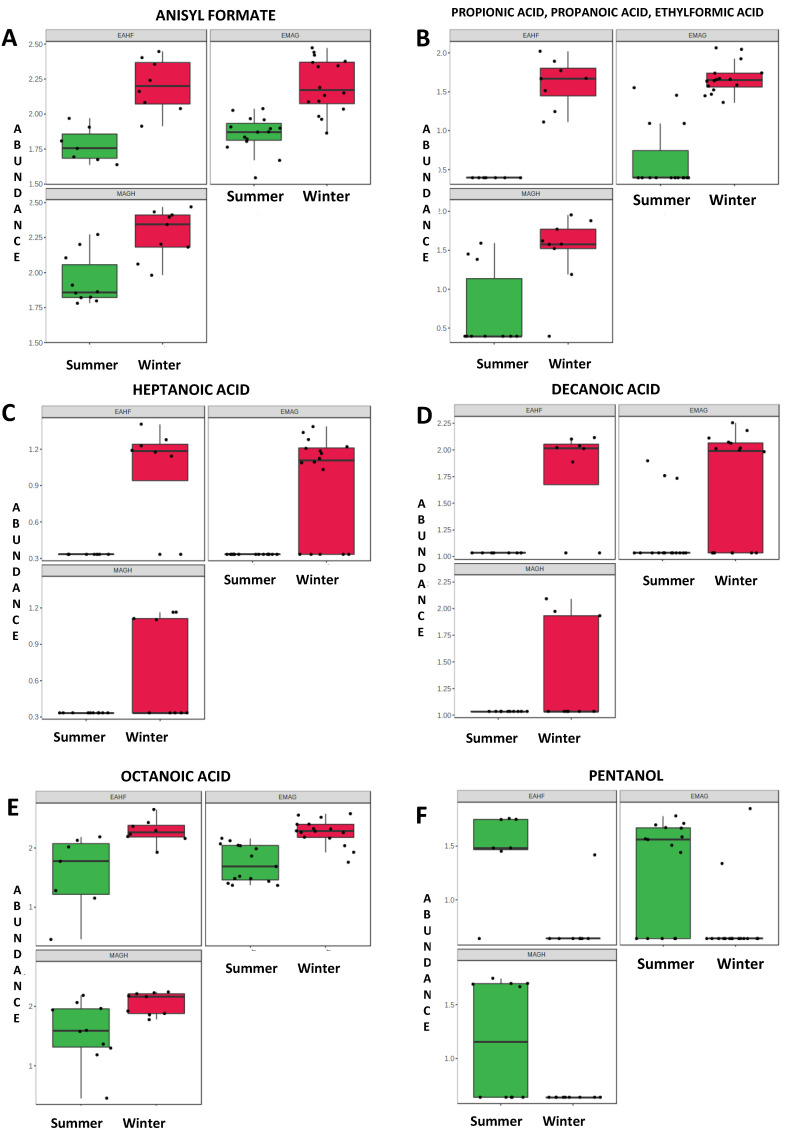
Bi-plot box-whisker of the raw milk volatile organic compounds (VOC) abundance (expressed as log_10_ of peak area) that were significantly (*p* < 0.05) affected by season (first group: anisyl formate, carboxylic acids and 2-pentanol). Feeding systems: EMAG, ensiled maize and grass; EAHF, ensiled and haylage forage; MAGH, meadow and grass hay. In each plot: median (bar in box), first and third quartile (bottom and top end of the box), min and max values (whiskers).

**Figure 2:**
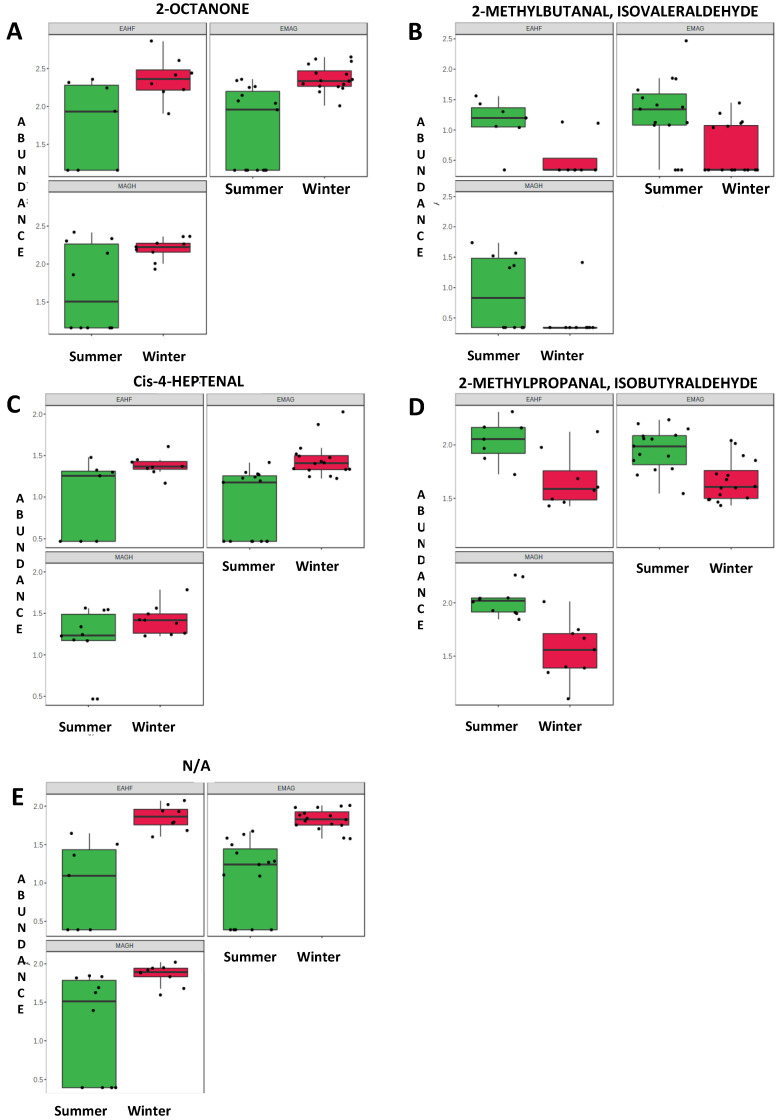
Bi-plot box-whisker of the raw milk volatile organic compounds (VOC) abundance (expressed as log_10_ of peak area) that were significantly (*p* < 0.05) affected by season (second group: 2-octanone, aldehydes and the unassigned compound). Feeding systems: EMAG, ensiled maize and grass; EAHF, ensiled and haylage forage; MAGH, meadow and grass hay. In each plot: median (bar in box), first and third quartile (bottom and top end of the box), min and max values (whiskers).

Two methyl aldehydes (2-methylproponal and 2-methylbutanal) were identified as biomarkers of the summer milk samples, which had also a higher content of 2-pentanol. The relative concentrations (as log_10_ of the peak area, abundance) of the main informative VOCs are also depicted in box plots, which emphasise the variability in the milk of heptanoic acid, decanoic acid, 2-pentanol (Figure 1), 2-octanone and cis-4 heptenal (Figure 2).

**Figure 3:**
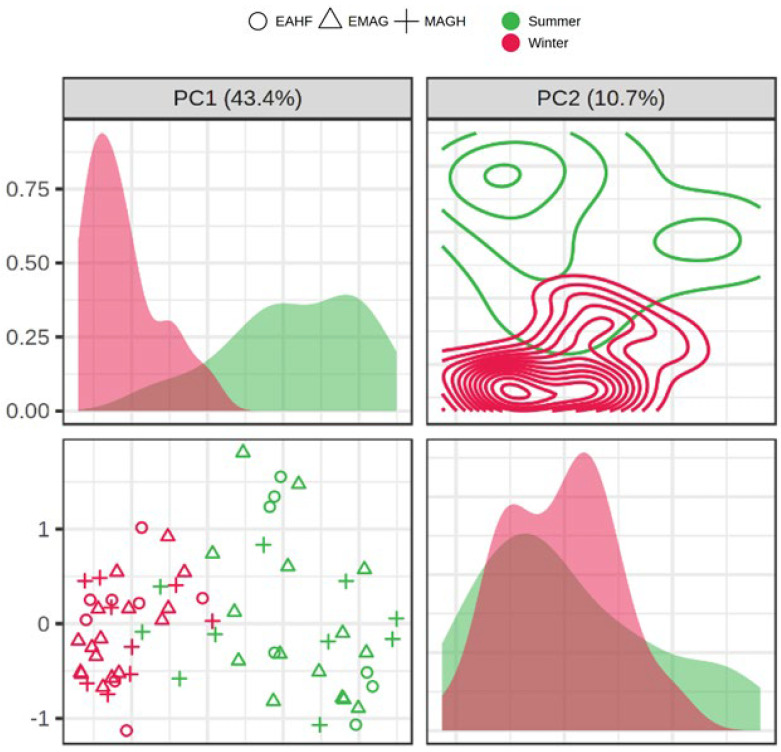
Interactive principal component analysis (iPCA) applied to the volatile organic compound (VOC) profiles of the raw milk according to the feeding systems (EMAG, ensiled maize and grass, Δ; EAHF, ensiled and haylage forage ○; MAGH, meadow and grass hay, +) and seasons (summer, green; winter, red).

The explorative IPCA displayed a limited capacity of the VOC profile to discriminate milk samples from the three feeding systems, whereas the graphical outcomes of the iPCA showed a clear spatial separation between the production season along both principal component 1 (PC1) and 2 (PC2) that explained 54.1% of the total variance (Figure 3). The outcomes of iPCA revealed a higher variability within summer milk samples compared to those produced in winter. 

## 4. Discussion

### 4.1. Milk Composition and Chemical Traits

In accordance with the outcomes of previous investigations [27,28], the lower amounts of protein and fat detected in summer milk were likely due to physiological and metabolic changes correlated to cows’ heat stress [29]. Under prolonged exposure to the warm temperatures, cow feeding behaviour is characterised by lower dry matter intake and higher consumption of water, with dilution of the rumen content and decreases of saliva production, rumen motility and bacteria activity. This, in turn, implies a reduction of nutrient digestibility, along with an imbalance between the degradation rate of carbohydrates and N-sources, impairing milk quality with less protein and fat synthesis [30]. The reduction of the energy to crude protein intake and the consequent lower metabolizable energy level that seemed to characterise the summer time is a feeding condition that coupled with a specific downregulation of mammary protein synthesis could explain the more undesirable effect in decreasing milk true protein and casein concentrations [31]. Compared with winter milk, the higher content of urea observed in the summer milk, which led to higher milk pH, could be also explained by a strengthening of skeletal muscle catabolism related to rising temperatures, which induces an increment of both plasma and milk urea nitrogen [32]. Furthermore, the seasonal changes in urea contents may be also correlated with a variation of the milk microbiome composition [33]. 

### 4.2. Milk Volatile Organic Compounds

Using an overview comprehensive analysis based on a triphasic DVB/CAR/PDMS fiber allowing the retrieval of both high and low polarity volatile compounds [7], and a non-targeted approach, the study investigated the differences in VOC profiles of raw milk produced in winter and summer. The studied farms belonged to dairy chains that produce high-value PDO cheeses by processing raw milk with mild technologies capable of preserving both the nutritional properties and the VOCs. To better capture the effect of the seasonality, besides that arising from metabolic differences caused by the dietary factors, milk samples from three year-round indoor FS that differed in the main forage sources included in the TMR of the lactating cows were investigated. A diverse and balanced sample set ensures the reliability of the volatile markers retrieved from the statistical modelling. Note that a non-targeted study based on a limited homogeneous sample set likely fails to capture the large-scale variance expected in food matrices and can lead to the retrieval of misleading molecular markers. Heterogeneous sets of samples and analytical repetitions enable: (i) the appropriate inclusion of biological variance, (ii) the minimization of unexpected instrumental variations and (iii) the encompassment of all of the variability of the raw milk linked to the different dietary intakes [34,35]. 

The outcomes of ANOVA and explorative iPCA of the present study emphasised how, across the FSs, the volatile profile of raw milk was affected by seasonality. Specifically, the VOC profile of the milk samples obtained by the HS-SPME combined with GC-FID technique was similar across FSs, even in the comparison between maize silage-based (EMAG) and hay-based (MAGH) TMRs. The iPCA spatially illustrates the interaction result with season and FS and clearly highlights the impact of seasonality on the VOC profile of milk, with seasonal changes VOCs that are independent of the feeding regime. The impact of annual season on raw milk’s VOC composition is still debated [4]. Only limited information is available on the seasonal change in milk’s VOC profile within a similar year-round indoor feeding system, and therefore, full understanding of the reasons for the different VOC pattern measured across winter and summer is challenging and beyond the scope of this research. A recent study carried out by Nalepa et al. (2018) [18] in north-eastern Poland showed a significant seasonal variation in raw milk bacterial microbiota and some correlations between selected microorganisms and clusters of VOCs, but no data about the feeding regimen were provided. However, relatively few details were given about VOC variations across the investigated period, except for a higher number of esters, alcohols and ketones in spring-summer and of free fatty acids (C2–C7) in autumn-winter. 

In accordance with the literature [18,36], the outcomes of the present study highlighted that winter milk had higher levels of C3-C10 carboxylic acids. The presence of octanoic and decanoic acids is probably due to their incomplete esterification in the mammary gland before lipid creation and/or the spontaneously lipolytic activity in milk from endogenous enzymes [37]. Previous studies on several milk samples from different cow breeds and feeding regimes showed that fatty acids (FA) profile was affected across milking seasons [38], with a significantly increase of saturated short- and medium-chain (FA 4:0 to 14:0) triacylglycerols in winter months [39]. Likewise in this study, it appeared that milk lipidomes were affected by both dietary and season conditions, which seemed to be partially overlapped over a full year [40]. 

With regards to anisyl formate and cis-4-heptenal, only the latter was previously detected in milk [41], but the origins of these compounds remain unclear. Moreover, 2-heptenal (or its isomer) has been previously identified as a biomarker of raw milk from TMR-based feeding systems [12]. As methyl ketones can be derived by β-oxidation of saturated fatty acids, 2-octanone in our winter milk could be correlated with the significantly higher amount of milk fat observed in the cooler season as suggested by previous findings [42]. The main metabolic route for the transfer of forage and feed VOCs to the milk is their absorption in the digestive tract, but they could also be transferred to the mammary gland via blood stream after pulmonary inhalation or rumen gas production [10]. This second metabolic route might contribute to explain the differences measured in the current study of the raw milk VOC profiles between the two milking seasons.

Both methyl aldehydes (2-methylproponal and 2-methylbutanal), measured in quantities making them suitable as biomarkers for summer milk, were likely produced by degradation of amino acids rather than by lipid oxidation [7]. They could originate from formation in forage and feed during the storage period [43]. The high content of 2-pentanol in summer milk could be argued as secondary products of early endogenous milk lipolysis and further oxidation [9], although it could also originate from the metabolic activity of rumen microbial populations [10].

Forages included in the three indoor feeding systems were mainly winter cereals, and grass and maize that were harvested and ensiled from late spring to late summer to be stored long-term and used over the following year for cattle feed [44]. Low winter temperatures ensure the aerobic stability while silage fermentations resume as weather warms up in spring due to enterobacteria and yeasts. The former ferment available sugars, producing mainly acetic acid, with less quantities of ethanol, formic acid, 2,3-butanediol and CO_2_; yeasts ferment sugars to mainly ethanol and CO_2_ along with small amounts of other alcohols and volatile fatty acids [45]. Furthermore, the activity of some polyphenol oxidase of both ensiled or dried grass and legume forages, which exerts a positive role in protecting lipids from endogenous lipolytic activity, seemed to be related to the forage storage period [46]. Such changes in forage chemical composition could affect either cows’ feeding behaviour or the metabolism of carbohydrates and protein in the rumen. This could be the origin of the different native macro- and micro-compounds detected in the milk in winter compared to summer [47]. Moreover, evidence has been reported of a strong impact of raw milk’s indigenous microbial community on the development of its VOC profile, but indoor milk is more likely to have higher proportions of host/gut-associated microbes [48].

Overall, the outcomes of this study suggested that while there is no impact of forage types, milking season significantly affected raw milk VOC profile. Therefore, the investigation of this potential impact on sensory properties is crucial since it could alter the aroma perception of dairy derivates (e.g., fermented or curdled milks) processed using raw milk. It is noteworthy that when differences exist they are often difficult to be detected also by trained assessors [49], and yet perceived by consumers. Therefore, the use of GC and/or other olfactometry techniques is a useful operative approach to identify aroma-active VOCs capable of positively or negatively exacerbating sensory quality when selecting raw milk for further processing through mild technologies. 

## 5. Conclusions

The study pointed out that the indoor feeding system did not affect raw milk’s VOC profile, even when milk samples from cows fed TMRs based on ensiled forages or on permanent meadow hay were compared. In contrast, regardless of the cows’ feeding system, a significant seasonal effect on milk’s VOC profile was measured. Winter milk had higher proportions of carboxylic acids, while, in summer, milk was enriched with 2-pentanol and deprived of methyl ketones. Specific branched aldehydes contributed to discriminating the VOC profile of summer milks. With specific reference to intensive indoor milk production systems, the main outcome of this study is that VOC profile should be considered when proposing a fingerprinting approach for the origin assessment and traceability of labelled dairy products, especially for those obtained by the processing of raw milk with mild technologies. It must be emphasised that the operative recommendations provided in this manuscript aim to enhance the control of harvesting and farm storage conditions of both ensiled and dried forages since their dietary inclusion across the entire lactation can alter the presence and/or abundance of some aroma-active compounds impacting consumer preference. 

## Figures and Tables

**Table 1:** Averaged (±s.d.) values of ingredients (% on DM basis) and main chemical traits (% on DM basis) of the total mixed rations used in the three indoor feeding systems over two milk production seasons.

	EMAG (Ensiled Forage)		EAHF (Ensiled/Haylage Forage)		MAGH (Dried Forage) ^1^	
	Winter (n = 16)	Summer (n = 15)	Winter (n = 9)	Summer (n = 10)	Winter (n = 8)	Summer (n = 8)
Ingredients ^2^						
Maize silage (%)	35 (±6)	28 (±6)	11 (±3)	8 (±2)	0	0
Winter cereal silage (%)	6 (±2)	8 (±2)	17 (±7)	16 (±7)	0	0
Haylage (%)	2 (±1)	3 (±1)	13 (±4)	10 (±3)	12 (±2)	11 (±3)
Fresh herbage (%)	0	0	0	9 (±2)	0	0
Hay (%)	10 (±3)	11 (±3)	10 (±2)	7 (±2)	36 (±4)	37 (±5)
Concentrates (%)	43 (±4)	45 (±4)	45 (±6)	45 (±6)	47 (±3)	45 (±4)
Residual (%)	4 (±2)	5 (±2)	4 (±2)	5 (±2)	5 (±1)	7 (±1)
Chemical traits ^3^						
Crude protein (%)	14.6 (±0.5)	14.5 (±0.6)	14.3 (±0.7)	14.5 (±0.5)	13.9 (±0.8)	14.1 (±0.8)
aNDF (%)	35.8 (±2.7)	35.6 (±2.6)	35.9 (±2.7)	36.8 (±3.5)	38.2 (±4.4)	38.6 (±4.2)
ADF	22.1 (±1.4)	21.9 (±1.4)	21.8 (±1.7)	22.6 (±2.0)	23.4 (±2.2)	23.8 (±2.4)
ADL	3.3 (±0.3)	3.5 (±0.3)	3.6 (±0.4)	3.5 (±0.5)	3.8 (±0.3)	4.1 (±0.5)
NFC (%)	38.5 (±2.2)	38.9 (±2.3)	38.3 (±2.5)	37.5 (±2.6)	37.4 (±3.2)	37.0 (±2.9)
Starch (%)	24.4 (±2.2)	24.6 (±2.1)	23.3 (±2.5)	23.1 (±2.4)	20.9 (±2.4)	20.5 (±2.5)
aNDF/starch	1.47	1.45	1.54	1.59	1.83	1.88

^1^ EMAG, ensiled maize and grass; EAHF, ensiled and haylage forage; MAGH, meadow and grass hay. ^2^ Haylage and fresh herbage from permanent meadow and perennial ryegrass; hay from permanent meadow and lucerne; concentrates comprised maize, soybean, barley, sunflower and wheat products; residual comprised straw, bran, beet pulps, molasses and vitamin-mineral mix. ^3^ aNDF, (amylase) neutral detergent fiber; ADF, acid detergent fiber; ADL, acid detergent lignin (sulphuric acid lignin); NFC, non-fiber carbohydrates.

**Table 2:** Effect of feeding system (FS) and season on main constituents of raw milk.

Item ^1^	Feeding System ^2^				Season			RMSE ^3^
	EMAG	EAHF	MAGH	*p*-Value	Winter	Summer	*p*-Value	
Protein, g/100 g	3.39 ^b^	3.38 ^b^	3.48 ^a^	0.003	3.45 ^a^	3.38 ^b^	0.012	0.182
Casein, g/100 g	2.58 ^b^	2.60 ^b^	2.68 ^a^	0.008	2.70 ^a^	2.57 ^b^	0.009	0.159
Fat, g/100 g	4.00	3.96	3.95	0.737	4.07 ^a^	3.87 ^b^	0.003	0.223
Lactose, g/100 g	4.82 ^a^	4.80 ^a^	4.72 ^b^	0.001	4.79	4.76	0.123	0.073
SCS, U	3.6	3.7	4.1	0.103	3.8	3.8	0.874	0.573
pH	6.64	6.66	6.65	0.144	6.62 ^b^	6.68 ^a^	0.011	0.020
β-HB, mmol/L	0.070	0.067	0.068	0.924	0.067	0.070	0.692	0.031
Urea (mg/dL)	20.7	23.8	23.7	0.150	20.9 ^b^	24.6 ^a^	0.013	3.523

^1^ β-HB, β-hydroxybutyrate; SCS, somatic cell score as [log_2_ (SCC/100,000) + 3]. ^2^ EMAG, ensiled maize and grass; EAHF, ensiled and haylage forage; MAGH, meadow and grass hay. ^3^ RMSE, root mean square error. Within feeding system and/or season, means with different superscript letters (^a,b^) in the same row differ by *p* < 0.05.

**Table 3:** Effect of the milking season on raw milk volatile organic compounds (VOCs).

**Retention Time (min)**	**Name**	**Class**	**Formula**	**Mass**	***p*-Value ^1^**
		Winter			
1.88	Propionic acid	Carboxylic acid	C_3_H_6_O_2_	74.079	0.001
4.25	cis-4-Heptenal	Aldehyde	C_7_H_12_O	112.172	0.019
6.51	2-Octanone	Ketone	C_8_H_16_O	128.215	0.008
9.48	Heptanoic acid	Carboxylic acid	C_7_H_14_O_2_	130.187	0.001
13.63	Octanoic acid	Carboxylic acid	C_8_H_16_O_2_	144.214	0.001
20.39	Anisyl formate	Aldehyde	C_9_H_10_O_3_	166.176	0.001
22.06	Decanoic acid	Carboxylic acid	C_10_H_20_O_2_	172.268	0.001
27.68	Not assigned				
		Summer			
1.43	2-methylpropanal	Aldehyde	C_4_H_8_O	72.107	0.002
1.65	2-methylbutanal	Aldehyde	C_5_H_10_O	86.134	0.022
1.96	2-Pentanol	Alcohol	C_5_H_12_O	88.15	0.001

^1^ *p*-value refers to the significance of the milk production season.

## Data Availability

The data presented in this research are available on request from the corresponding author.

## Supplementary Materials

The following supporting information can be downloaded at: https://www.mdpi.com/article/10.3390/foods12091871/s1, Table S1: The entire milk volatile organic compound profile according to the retention time (RT, min). Figure S1. Interactive principal component analysis (iPCA) applied to raw milk VOC profiles according to the feeding systems (EMAG, ensiled maize and grass, D; EAHF, ensiled and haylage forage, ○; MAGH, meadow and grass hay, +) and seasons (summer, green; winter, red). The principal components PC1 and PC2 explain the 54.3% of total variance. Contribution to the explanation of the variance was provided also by PC1-PC3 and PC1-PC4.

## Abbreviations

ADF	acid detergent fiber
ADL	acid detergent lignin
ANOVA	analysis of variance
AOAC	association of official analytical chemists
EHAF	ensiled and haylage forage
EMAG	ensiled maize and grass
DIM	days in milk
DM	dry matter
DVB/CAR/PDMS	divinylbenzene/carboxen/polydimethylsiloxane
FA	fatty acid
FID	flame ionization detector
FPCM	fat protein corrected milk
FS	feeding system
FT-MIR	Fourier-transform mid infrared
GC	gas chromatography
GC-FID	gas chromatography-flame ionization detection
β-HB	β-hydroxybutyrate
HS-SPME	headspace solid-phase microextraction
iPCA	interactive principal component analysis
IRMS	isotope ratio mass spectrometry
LC/MS	liquid chromatography/mass spectrometry
MAGH	meadow and grass hay
aNDF	(amylase) neutral detergent fiber
NFC	non-fiber carbohydrates
NIST	National Institute of Standards and Technology
NMR	nuclear magnetic resonance
PC	principal component
PDO	protected designation of origin
PTFE	polytetrafluoroethylene
RMSE	root mean square error
RTL	retention time locked
S	season
SCC	somatic cell count
SCS	somatic cell score
TMR	total mixed ration
VOC	volatile organic compound
