# Supplementary material for: Seasonal Variation in Raw Milk VOC Profile within Intensive Feeding Systems
Source: Foods. 2023 Apr 30;12(9):1871. doi: 10.3390/foods12091871 (PMC10178752; doi:10.3390/foods12091871)
Supplement: Supplementary file 1 [file foods-12-01871-s001.zip › foods-2343976-supplementary.pdf]

Supporting information

Table S1. The entire milk volatile organic compound profile according to the retention time (RT, min)

RT	Assignment	Chemical class	Formula	Molecular weight
1.43	Isobutyraldehyde	ALDEHYDE	C_4H_8O	72.11
1.50	Isopropyl alcohol; Isopropanol	ALCOHOL	$C3H_8O$	60.10
1.56	Diacetyl	KETONE	$C_4H_6O_2$	86.09
1.61	Hexane	ALKANE	C_6H_{14}	86.18
1.62	N/A	-	-	-
1.65	3-Methylbutanal; Isovaleraldehyde	ALDEHYDE	$C_5H_{10}O$	86.13
1.72	isobutyl alcohol	ALCOHOL	$C_4H_{10}O$	74.123
1.88	Propionic acid, Propanoic acid, Ethylformic acid	CARBOXYLIC ACID	$C_3H_6O_2$	74.08
1.96	2-Pentanol	ALCOHOL	$C_5H_{12}O$	88.15
2.09	N/A	-	-	-
2.20	Isobutyric acid	CARBOXYLIC ACID	$C_4H_8O_2$	88.11
2.47	Amyl alcohol, 1-Pentanol	ALCOHOL	$C_5H_{12}O$	88.15
2.54	Isobutyl acetate	ESTER	$C_6H_{12}O_2$	116.16
2.64	Methyl-2-methylbutyrate	ESTER	$C_6H_{12}O_2$	116.16
2.81	3,4-Hexanedione	KETONE	$C_6H_{10}O_2$	114.07
3.05	Ethyl lactate	ESTER	$C_5H_{10}O_3$	118.13
3.68	2-Hexen-1-ol (trans)	ALCOHOL	$C_6H_{12}O$	100.16
4.17	Valeric acid	CARBOXYLIC ACID	$C_5H_{10}O_2$	102.13
4.25	cis-4-Heptenal	ALDEHYDE	$C_7H_{12}O$	112.17
4.35	Ethyl valerate; Ethyl pentanoate	ESTER	$C_7H_{14}O_2$	130.18
4.45	Heptanal	ALDEHYDE	$C_7H_{14}O$	114.10
4.77	Isobutyl isobutyrate	ESTER	$C_8H_{16}O_2$	144.21
6.35	Hexanoic acid	CARBOXYLIC ACID	$C_6H_{12}O_2$	117.15
6.51	2-Octanone	KETONE	$C_8H_{16}O$	128.215
6.84	2-Octanol	ALCOHOL	$C_8H_{18}O$	130.23
7.60	p-Methylanisole	ETHER	$C_8H_{10}O$	122.17
7.87	2-Ethyl-1-hexanol	ALCOHOL	$C_8H_{18}O$	130.23
8.01	Isopropyl hexanoate	ESTER	$C_9H_{18}O_2$	158.24
9.48	Heptanoic acid	CARBOXYLIC ACID	$C_7H_{14}O_2$	130.19
9.64	Butyl butyrate	ESTER	$C_8H_{16}O_2$	144.21
10.29	Methyl benzoate	ESTER	$C_8H_8O_2$	136.15
11.15	Isophorone; 3, 5, 5-Trimethyl-2-cyclohexen-1-one	KETONE	$C_9H_{14}O$	138.21
12.80	alpha,alpha-Dimethylphenethyl acetate	ESTER	$C_{12}H_{16}O_2$	192.26
13.61	Diethyl succinate	ESTER	$C_8H_{14}O_4$	174.20
13.63	Octanoic acid	CARBOXYLIC ACID	$C_8H_{16}O_2$	144.214

15.13	trans,trans-2,4-Nonadienal	ALDEHYDE	$C_9H_{14}O$	138.21
16.12	cis-3-Hexenyl isovalerate, Hex-3-enyl isovalerate	ESTER	$C_{11}H_{20}O_2$	184.28
20.39	Anisyl formate	ALDEHYDE	$C_9H_{10}O_3$	166.18
21.95	3-Phenylpropyl acetate	ESTER	$C_{11}H_{14}O_2$	178.23
22.06	Decanoic acid	CARBOXYLIC ACID	$C_{10}H_{20}O_2$	172.27
24.35	trans-Cinnamic acid	CARBOXYLIC ACID	$C_9H_8O_2$	148.16
27.68	N/A	-	-	-
34.59	gamma-Dodecalactone	KETONE	$C_{12}H_{22}O_2$	198.306

N/A, not assigned

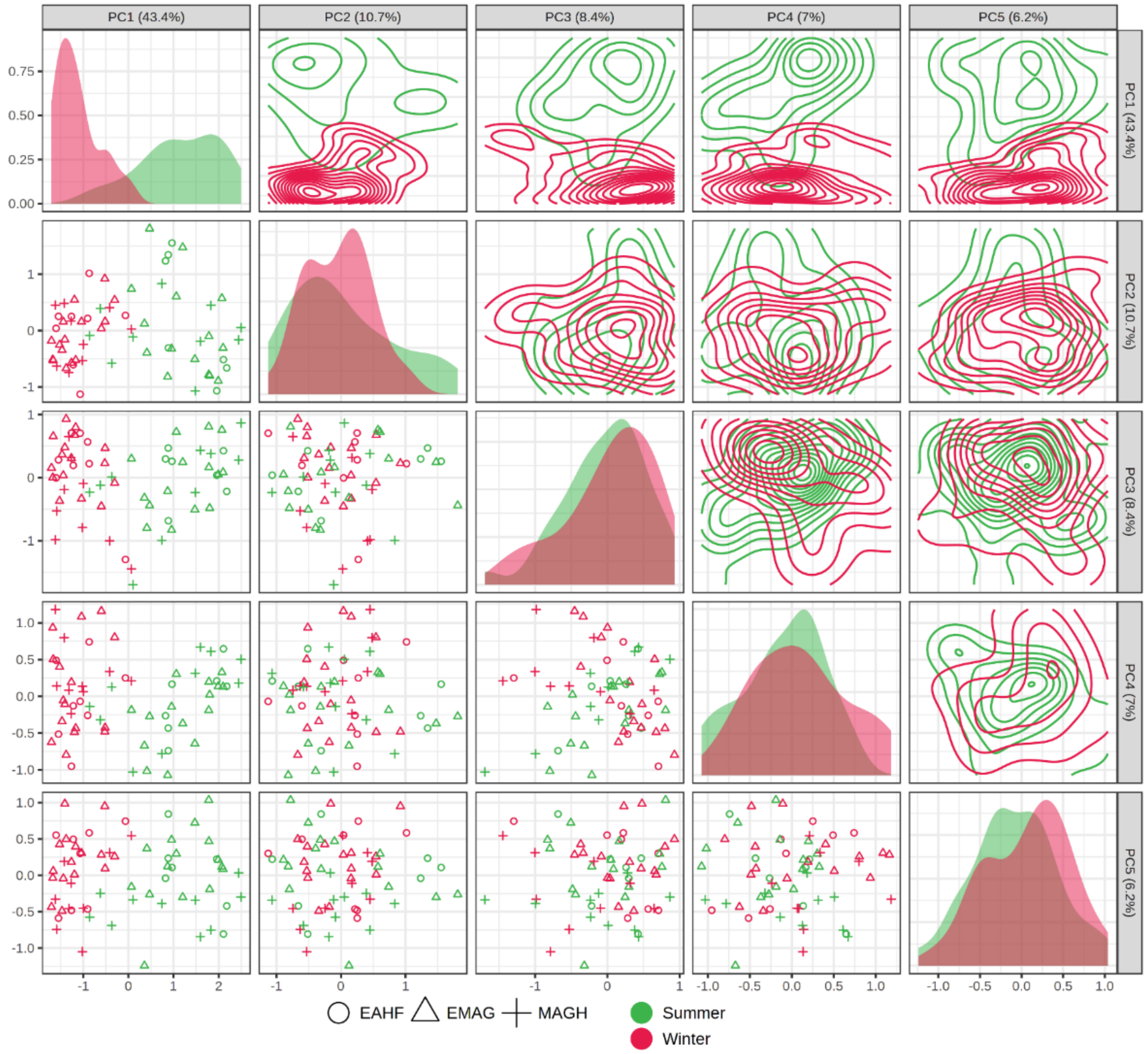

Figure S1. Interactive principal component analysis (iPCA) applied to raw milk VOC profiles according to the feeding systems (EMAG, ensiled maize and grass, Δ; EAHF, ensiled and haylage forage, ○; MAGH, meadow and grass hay, +) and seasons (summer, green; winter, red). The principal components PC1 and PC2 explain the 54.3% of total variance. Contribution to the explanation of the variance was provided also by PC1-PC3 and PC1-PC4.
